# The effects of functionally monocular patients’ emotional reactions during phacoemulsification under topical anesthesia

**DOI:** 10.5935/0004-2749.20210014

**Published:** 2025-02-02

**Authors:** Paula Kataguiri, Marina Paulino Gracia, Guilherme Murrer, Aline Sutili Toledo, José Ricardo C. L. Rehder, Vagner Loduca, Newton Kara-Junior

**Affiliations:** 1 Department of Ophthalmology, Faculdade de Medicina do ABC, Santo André, SP, Brazil; 2 Department of Ophthalmology, Universidade de São Paulo, São Paulo, SP, Brazil

**Keywords:** Phacoemulsification/psychology, Capsulorhexis, Anesthestics, local, Visual acuity, Facoemulsificação/psicologia, Capsulorrexe, Anestésicos locais, Acuidade visual

## Abstract

**Purpose:**

To evaluate the relationship between the incidence of complications and functionally monocular patients’ emotional reactions during phacoemulsification under topical anesthesia.

**Methods:**

We enrolled 22 functionally monocular patients (11 males and 11 females; group 1) and 19 ageand sex-matched controls (6 males and 13 females; group 2) in this prospective, interventional, cross-sectional, case control study. Demographics data, including age, sex, and educational background, were collected. Surgeries were performed by the same surgeon, and during surgery, the patients’ vital signs (blood pressure and heart rate) and surgical events (duration, body movements, signs of increased vitreous cavity pressure, difficulty in performing capsulorhexis, and complications) were noted. Preand postoperative visual acuity was also analyzed.

**Results:**

The mean age of group 1 was 73.05 ± 13.31 years and of group 1 was 69.74 ± 16.81 years. There was no significant between-group difference in systolic and diastolic blood pressures. The average heart rate was similar in both groups, too. During surgery, the surgeon’s perception of excessive eye, eyelid, or head movements in both groups was similar, in addition to signs of increased vitreous cavity pressure.

**Conclusion:**

It is safe to perform phacoemulsification under topical anesthesia in functionally monocular patients, who apparently behave similarly to binocular patients.

## Abstract (pt)

**Objetivo:**

Avaliar a relação entre a incidência de complicações e reações emocionais durante a cirurgia de cat arata sob anestesia tópica em pacientes funcionalmente monoculares.

**Métodos:**

Estudo prospectivo, transversal, caso-controle de vinte e dois pacientes monoculares e dezenove controles pareados por idade e sexo . Dados demográficos foram analisados: idade, sexo e escolaridade. As cirurgias foram realizadas pelo mesmo cirurgião e durante o procedimento os sinais vitais dos pacientes (como pressão arterial sistêmica e frequência cardíaca) e eventos cirúrgicos (duração da cirurgia, movimentos corporais, sinais de aumento da pressão vítrea, dificuldade de realização da capsulorrexis e complicações) foram coletados. A acuidade visual pré e pós foi analisada. A distribuição normal dos dados foi confirmada com o teste de Shapiro-Wilk. Os dados foram expressos como média ± DP e porcentagem. A comparação dos diferentes testes clínicos entre os grupos foi realizada utilizando Student’s t-test e ANOVA com correção de Bonferroni. O qui-quadrado foi usado para comparar dados demográficos. Valor de p<0,05 foi considerado estatisticamente significante.

**Resultados:**

Este estudo incluiu vinte e dois olhos de 22 pacientes funcionalmente monoculares (6 homens e 13 mulheres) e dezenove olhos de 19 controles (11 homens e 11 mulheres). A média de idade foi de 73,05 ± 13,31 anos nos indivíduos monoculares e 69,74 ± 16,81 no controle. Considerando-se os sinais vitais não houve diferença significativa entre os grupos (p>0,05). Durante o procedimento, a percepção do cirurgião em relação aos movimentos excessivos de olho, pálpebra ou cabeça em ambos os grupos foi semelhante, assim como sinais de aumento da pressão vítrea (p=0,2 e p=0,1, respectivamente).

**Conclusão:**

Este estudo sugere que é seguro realizar a extração de catarata com anestesia tópica em pacientes funcionalmente monoculares. Esses pacientes aparentemente se comportam de maneira semelhante aos pacientes binoculares.

## INTRODUCTION

Blindness is a major public health issue^(1,2)^. In the event of severe vision loss in one eye, the patient is considered monocular if Snellen best-corrected visual acuity (BCVA) in the other eye is worse than 20/200^(3,4)^.

Cataracts are responsible for approximately 50% of blindness cases in Brazil^(5)^. Ophthalmologists, in general, are reluctant to recommend surgery if a patient with one blind eye develops a cataract in the other eye because any surgical complication might cause blindness^(6)^. On the other hand, monocular patients may delay necessary surgery for fear of surgical risks and become blind as a result^(4)^.

Phacoemulsification is usually performed with the patient is awake, and topical anesthesia is the most frequent choice of experienced surgeons^(7)^. In monocular patients, anxiety manifestations, such as uncontrolled head or eye movements, might make the surgery more difficult and increase the incidence of complications^(3,8,9)^. Fear and anxiety are higher in monocular patients when phacoemulsification is recommended^(10,11)^. However, there is little information available on the effects of such emotional factors during surgery. This study evaluated the possible relationship between the incidence of complications and functionally monocular patients’ emotional reactions during phacoemulsification under topical anesthesia.

## METHODS

### Patients and procedure

This prospective, interventional, cross-sectional case control study was approved by the Universidade de São Paulo Ethics Committee according to the tenets of the Declaration of Helsinki, and the study protocol was approved by the University’s Institutional Review Board. We enrolled 41 patients, aged ≥40 years, consecutively screened for phacoemulsification in a tertiary referral center in São Paulo, Brazil between 2011 and 2012. The inclusion criterion was the absence of any eye alteration besides senile cataract in the eye to be operated on. Patients who could not be sedated and, thus, had to be given peribulbar block or general anesthesia were excluded. All patients willingly participated in the study. Patients were considered monocular via diagnosis of irreversible blindness in the contralateral eye, that is, BCVA <=20/200 according to Snellen chart or visual field <20°^(8)^. Patients were considered binocular if both eyes, despite crystalline opacity, had potential vision and there was the possibility of significant visual acuity improvement after phacoemulsification^(5)^.

Patients were divided into two groups: group 1 comprised 22 monocular individuals who were to undergo phacoemulsification in the only functional eye, and group 2 comprised 19 binocular individuals who were to undergo for the first time phacoemulsification in the eye with lower acuity.

All patients underwent a complete ophthalmologic examination with refractometry, applanation tonometry, biomicroscopy, and fundoscopy. Their vital signs were checked on admission (baseline) and 5 min after the beginning of surgery. Systolic blood pressure (SBP), diastolic blood pressure (DBP), and heart rate (HR) were measured by an experienced nurse, using an electric sphygmomanometer, and these parameters were used as objective measurements of anxiety, as previously described^(6)^. After surgery, a trained investigator administered a questionnaire to the surgeon in order to evaluate any difficulties he encountered, any intraoperative complications, and whether the patients’ emotional state influenced the procedure (Attachment 1). During surgery, the surgeon registered signs of increased vitreous cavity pressure whenever they occurred. Such signs were characterized by at least one of the following manifestations: decreased anterior chamber depth, iris prolapse out of the incision, excessive surge during emulsification of nuclear fragments, and difficulty with implanting the intraocular lens.

**Attachment 1:** 

Difficulty in performing capsulorhexis? Yes/No
Eye, head, or eyelid movements? Yes/No
Signs of increased vitreous cavity pressure? Yes/No
Complications? Yes/No. If yes:
4a. Zonular dehiscence
4b. Iris prolapse out of the incision
4c. Posterior capsule rupture without vitreous loss
4d. Posterior capsule rupture with vitreous loss
4e. Need for suture closure

All procedures were performed under topical anesthesia. A specialist was always present, who would lightly sedate each patient for relaxation without loss of consciousness if the surgeon noticed increased anxiety.

Before surgery, the surgeon subjectively evaluated each patient’s cooperative conditions for the procedure. Most of the patients in both groups agreed to undergo surgery under topical anesthesia without complementary sedation. All phacoemulsification procedures were performed by the same surgeon, using the AMO Sovereign phacoemulsification system (Irvine, California, USA) technique, with a 2.75 mm corneal incision, no sutures, and hydroxypropyl methylcellulose viscoelastic material (Ophthalmos, São Paulo, Brazil). The Rayner Adaptis intraocular lens (San Diego, CA, USA) was implanted.

### Statistical analyses

All statistical analyses were performed using SPSS Statistics 13.0 (SPSS Inc., Chicago, IL, USA). The normal data distribution was confirmed using the Shapiro-Wilk test. Data were expressed as mean ± standard deviation and percentage. Between-group clinical tests were compared using Student’s *t*-test and analysis of variance (ANOVA) with Bonferroni correction. The chi-square test was used to compare demographics data. P<0.05 was considered statistically significant.

## RESULTS

There were not statistically significant between-group differences in terms of age, gender, and educational background. There was no follow-up loss or exclusion of patients during the study. The mean age of group 1 was 73.05 ± 13.31 years and that of group 2 was 69.74 ± 16.81 years. There was no statistical between-group difference in the time between cataract diagnosis and surgery. The preoperative BCVA was also similar in both groups (p>0.05) (Table 1).

**Table 1:** Demographics

Characterists	Group 1	Group 2	p
	Monocular	Control	
Gender			
Female/male	50%/50%	68%/32%	>0.05
Educational background			
None	23%	11%	
Elementary	68%	53%	
Higher school	5%	16%	
Higher education	5%	21%	
Age (years) [mean/±SD]	73.05 ± 13.31	69.74 ± 16.81	>0.05
Mean preoperative VA (logMar)	0.94 ± 0.87	0.89 ± 0.76	>0.05

SD= standard deviation.

On the basis of the surgeon’s subjective evaluation, anesthetic sedation was required for six patients (22.27%) in group 1 and five patients (26.32%) in group 2 (p=0.763) because of anxiety manifestations, such as excessive eye or head movements, during surgery. The HR, SBP, and DBP were similar in both groups (p>0.05). The surgeon also observed similar levels of patient cooperation (similar eye, eyelid, and head movements) in both groups (p=0.790). In addition, the surgery duration was similar in both groups (Table 2). These findings suggested that the anxiety state manifested during surgery was similar in both groups of patients.

**Table 2:** Emotional factors

Characterists [mean/±SD]	Group 1	Group 2	P
	Monocular	Control	
Systolic blood pressure (mmHg)	182.11 ± 25.70	179.11 ± 23.03	0.698
Diastolic blood pressure (mmHg)	101.14 ± 24.60	98.37 ± 22.11	0.708
Heart rate (bpm)	74.36 ±11.20	75.32 ± 8.82	0.765

SD= standard deviation.

The surgeon reported difficulty in performing capsulorhexis in four patients (18.18%) in group 1 and one patient (5.26%) in group (p=0.2). Imperfect capsulorhexis, with extension of the flap to the periphery of the capsular bag, occurred in two patients in group 1 (9.09%). Signs of increased vitreous cavity pressure were found in three patients (13.64%) in group 1 and three patients in group 2 (15.79%) (p=0.846) (Table 3). In addition, five patients (22.73%) in group 1 and one patient (5.26%) in group 2 presented surgical complications (p=0.115).

**Table 3:** Surgery aspects and surgeon’s perception

	Group 1	Group 1	
**Characterists**	**Monocular**	**Monocular**	**p**
Time length of surgery (minutes) [mean/±SD]	9.18 ± 3.53	8.00 ± 2.44	0.228
Necessity of sedation	22%	26%	0.763
Difficulty in the capsulorhexis perfomance	18%	5%	0.207
Eye, head or eyelid movements	41%	37%	0.79
Signs of increased pressure in the vitreous cavity	14%	16%	0.846
Complications	23%	5%	0.115

y= mean; SD= standard deviation.

## DISCUSSION

Phacoemulsification is one of the most performed standard procedures worldwide because of fast visual recovery and low intraoperative complications. However, if complications do occur, they might compromise visual acuity in the operated eye^(12)^.

One advantage of phacoemulsification over extracapsular extraction of the crystalline lens is the fact that the former might be performed under topical anesthesia, with or without sedation, which decreases the risk related to anesthetic blockage and, therefore, clinically benefits patients^(13)^. However, during phacoemulsifi cation under topical anesthesia, difficulties and complications might arise because of emotional alterations, such as excessive fear and anxiety, resulting in occasional body movements or increased vitreous cavity pressure from excessive eyelid contraction^(13)^.

A patient’s vital signs, such as BP and HR, are influenced by his or her emotional factors. These should be measured throughout the phacoemulsification procedure to monitor the patient’s anxiety state.

Marback et al. (2010) reported that functionally monocular patients show increased anxiety, fear, and doubts with regard to surgery compared to binocular patients. During surgery, anxiety manifestations, such as excessive eye, eyelid, or head movements, and other manifestations, such as decreased anterior chamber depth, excessive surge during emulsification of nuclear fragments, iris prolapse, or difficulty with implanting the intraocular lens, are similar in patients. Surgeons should consider these emotional reactions and invest more time in explaining surgery-related risks and benefits to their patients^(4)^.

Marback et al.^(4)^ observed that, whenever phacoemulsification is indicated, functionally monocular patients take longer to decide whether to undergo the procedure compared to binocular patients^(11)^. The reason is probably the greater fear monocular patients present.

In cooperative patients, topical anesthesia is considered safer than the peribulbar technique^(8)^. Surgery under general anesthesia is only indicated in patients who lack comprehension skills, such as children and mentally impaired individuals. So far, there is no evidence that sedation or general anesthesia reduces surgical risks in functionally monocular patients. In a retrospective study, Trotter et al. observed a similarity in the incidence of surgical complications among monoand binocular patients during phacoemulsification in which different anesthesia techniques were used^(9)^.

This study had a couple of limitations. First, the sample size was small. Second was the subjectivity involved in observation of emotional reactions during surgery.

In conclusion, it is safe to perform phacoemulsification in functionally monocular patients under topical anes thesia. These patients apparently behave similarly to binocular patients.
